# Dendritic orientation and branching distinguish a class of multifunctional turtle spinal interneurons

**DOI:** 10.3389/fncir.2014.00136

**Published:** 2014-11-13

**Authors:** Jonathan R. Holmes, Ari Berkowitz

**Affiliations:** ^1^Department of Biology, University of OklahomaNorman, OK, USA; ^2^Cellular & Behavioral Neurobiology Graduate Program, University of OklahomaNorman, OK, USA

**Keywords:** spinal cord, dendrite, sholl analysis, locomotion, scratching, premotor, swimming

## Abstract

Spinal interneurons can integrate diverse propriospinal and supraspinal inputs that trigger or modulate locomotion and other limb movements. These synaptic inputs can occur on distal dendrites and yet must remain effective at the soma. Active dendritic conductances may amplify distal dendritic inputs, but appear to play a minimal role during scratching, at least. Another possibility is that spinal interneurons that integrate inputs on distal dendrites have unusually simple dendritic trees that effectively funnel current to the soma. We previously described a class of spinal interneurons, called transverse interneurons (or T neurons), in adult turtles. T neurons were defined as having dendrites that extend further in the transverse plane than rostrocaudally and a soma that extends further mediolaterally than rostrocaudally. T neurons are multifunctional, as they were activated during both swimming and scratching motor patterns. T neurons had higher peak firing rates and larger membrane potential oscillations during scratching than scratch-activated interneurons with different dendritic morphologies (“non-T” neurons). These characteristics make T neurons good candidates to play an important role in integrating diverse inputs and generating or relaying rhythmic motor patterns. Here, we quantitatively investigated additional dendritic morphological characteristics of T neurons as compared to non-T neurons. We found that T neurons have less total dendritic length, a greater proportion of dendritic length in primary dendrites, and dendrites that are oriented more mediolaterally. Thus, T neuron dendritic trees extend far mediolaterally, yet are unusually simple, which may help channel synaptic current from distal dendrites in the lateral and ventral funiculi to the soma. In combination with T neuron physiological properties, these dendritic properties may help integrate supraspinal and propriospinal inputs and generate and/or modulate rhythmic limb movements.

## Introduction

Defining neuronal types within the central nervous system is an important step to determine the composition and function of neuronal circuits. It has been difficult to delineate reliably morphological-physiological types of spinal cord interneurons in adult mammals (Jankowska, 1992, 2001; Jankowska and Edgley, 2010). For this and other reasons, many researchers have turned to studying spinal interneuron types and circuits in embryonic and early postnatal animals, which have fewer and simpler morphological types of interneurons that can often be identified by their early expression of combinations of particular transcription factors (Goulding, 2009; Grillner and Jessell, 2009; Kiehn, 2011). Ultimately, however, we also want to understand the composition and function of spinal cord circuits in adult limbed vertebrates.

Spinal cord limb-control circuits receive abundant and diverse inputs that trigger and modulate limb movements (Lemon, 2008). These inputs include propriospinal inputs as well as supraspinal descending inputs from several brain regions. Individual spinal interneurons can integrate inputs from a wide variety of sources (Jankowska, 1992, 2001; Alstermark et al., 2007), which often arrive via axons in the lateral funiculus and ventral funiculus and may contact distal dendrites of spinal interneurons. This raises the question of how these distal inputs can effectively trigger or modulate spinal interneuron activity.

We have identified a morphological-physiological class of spinal interneurons, called transverse interneurons (or T neurons), that are good candidates to contribute importantly to the rhythmic activity of limb motoneurons during locomotion and scratching in adult turtles (Berkowitz et al., 2006). Turtles have the advantages of unusual resistance to hypoxia (Hounsgaard and Nicholson, 1990; Lutz and Milton, 2004) and a somewhat simpler spinal cord than adult mammals (Kusuma et al., 1979; Nissen et al., 2008). Also, the spinal cord of an adult turtle can appropriately generate the motor patterns for a variety of hind limb movements, including forward swimming, three forms of scratching, and flexion reflex, even without input from the brain, movement-related sensory feedback, and pharmacological manipulation (Stein, 2005). The spinal cord is largely conserved evolutionarily (Nieuwenhuys, 1964; Kusuma et al., 1979; Fetcho, 1992), so findings from the turtle spinal cord are likely to apply to mammals as well.

T neurons were defined morphologically, as interneurons having dendrites that extend further within the transverse plane (i.e., mediolaterally and dorsoventrally) than rostrocaudally and having mediolaterally elongated somata. T neurons were consistently activated during both scratching and swimming motor patterns and usually were activated during flexion reflex motor patterns as well (Berkowitz et al., 2006; Berkowitz, 2008). Thus, T neurons are multifunctional. T neurons also displayed a suite of electrophysiological characteristics correlated with their morphology (Berkowitz et al., 2006). Electrophysiological properties of T neurons were compared to those of scratch-activated spinal interneurons having quite different dendritic morphologies (which were called “non-T neurons” for convenience). T neurons reached higher peak firing rates and had larger membrane potential oscillations during scratching motor patterns than non-T neurons, suggesting an important role in rhythmic limb movement control.

Because T neurons typically have dendrites that extend far into the ventral and lateral funiculi, even up to the lateral edge of the spinal cord (Berkowitz et al., 2006), they are well positioned to receive synaptic inputs from a variety of supraspinal and propriospinal axons. Propriospinal axons, at least, make putative en passant synapses in the lateral funiculus in addition to terminal arbors in the gray matter (Berkowitz and Stein, 1994). But distally generated postsynaptic potentials would be greatly attenuated at the soma unless dendritic characteristics opposed such attenuation (Segev and London, 2000; Williams and Stuart, 2002, 2003; Gulledge et al., 2005). One possibility is that the dendrites have active conductances that maintain or amplify synaptic inputs (Gulledge et al., 2005; Johnston and Narayanan, 2008; Larkum and Nevian, 2008). Mammalian motoneurons (Heckman et al., 2008), turtle motoneurons (Hounsgaard and Kiehn, 1993) and probably some turtle ventral horn interneurons (Hounsgaard and Kjaerulff, 1992) can express active dendritic conductances. However, the intrinsic properties of spinal motoneurons, at least, probably play only a minor role during scratching due to overwhelmingly large rhythmic synaptic inputs (Alaburda et al., 2005).

Another possibility is that the dendritic trees of T neurons are unusually simple, so that current is constrained to flow along a relatively direct path to the soma and is thus more effective. It appeared subjectively from the earlier study that T neuron dendrites branched less and were oriented mediolaterally more often than rostrocaudally, but these dendritic properties were not assessed quantitatively or objectively (Berkowitz et al., 2006). Here, we investigate these apparent morphological characteristics quantitatively and systematically, using the same dataset of 17 T neurons and 14 non-T neurons as in the original study. We find that T neurons (compared to non-T neurons) have a significantly shorter total dendritic length, a significantly greater proportion of total dendritic length in primary dendrites, and a mean dendritic orientation that is significantly more mediolateral. These dendritic features may increase the effectiveness of distal inputs from synapses in the lateral funiculus and ventral funiculus. These findings expand the suite of morphological and physiological characteristics associated with this class of spinal interneurons, which likely contributes to many or all types of hind limb movements.

## Materials and methods

### Dataset

We carried out quantitative morphological analyses on the dendrites of the 17 T neurons and 14 non-T neurons initially described in an earlier study (Berkowitz et al., 2006). Briefly, each interneuron had been filled by current injection from an electrode containing 4% Neurobiotin, following characterization during scratching motor patterns in spinal cord-transected, immobilized, and artificially ventilated adult red-eared turtles (*Trachemys scripta elegans*). Under deep pentobarbital anesthesia, animals were perfused with saline followed by 4% paraformaldehyde. The spinal cord was frozen sectioned horizontally at 100 µm. Sections were reacted with avidin-biotin-horseradish peroxidase followed by SG reagent (Vector Laboratories) to reveal the labeled cells. Labeled neurons were reconstructed using a camera lucida. T neurons were defined as having a rostrocaudal/(mediolateral + dorsoventral) dendritic length ratio of <0.4 and a rostrocaudal/mediolateral soma length ratio of ≤1.0. Non-T neurons, a heterogeneous grouping of convenience for this comparison, were defined as having a dendritic length ratio of >0.5. All procedures in this previous study were approved by the Institutional Animal Care and Use Committee of the University of Oklahoma.

### Morphological analyses

ImageJ (National Institutes of Health), NeuronJ (Erik Meijering), and Excel (Microsoft) were used for all dendritic measurements. First, a scan of each neuron’s two-dimensional reconstruction (in the horizontal plane, with the dorsoventral axis collapsed; black on a white background) was inverted in Photoshop (Adobe Systems) to white on a black background. The Photoshop Sharpen function was used to eliminate gray pixels adjacent to the white lines of the dendrites. The micrometer scale bar from each reconstruction was used to calibrate the pixel-to-µm ratio and construct a scale bar in ImageJ.

Dendritic tracings were performed in NeuronJ, with Hessian smoothing scale at 4.0, cost weight factor at 0.9, tracing smoothing factor at 0, and a tracing subsampling factor of 5. Each dendritic branch of each reconstructed neuron was separately traced and each such tracing was labeled according to whether it was a primary dendrite, secondary dendrite, etc. A primary dendrite was defined as any branch emerging from the soma, a secondary dendrite as any branch emerging from a primary dendrite, etc., without regard to length or thickness of each branch, to avoid subjective biases. The NeuronJ Measure function was used to determine the length of each dendritic segment in each neuron. The .ndf file from the NeuronJ tracings of each neuron was exported to Excel. This file provided the x and y coordinates of each vertex of each tracing.

In Excel, these coordinates were used to measure the angle of the line segment between each adjacent pair of vertices along each dendritic branch with respect to the horizontal in these horizontal sections (e.g., an exactly mediolaterally oriented dendritic segment had an angle of 0°; an exactly rostrocaudally oriented dendritic segment had an angle of 90°). The mean dendritic angle for each neuron was calculated in Excel as the mean angle of all the dendritic line segments, with each segment’s angle value weighted by its proportional contribution to the total dendritic length.

For the Sholl analysis (number of dendritic branch intersections with concentric circles of increasing radii from the soma), the dendritic tracings from NeuronJ were saved as black lines on a white background. The Advanced Sholl Analysis tool of ImageJ was then applied, with a step size of 10 µm, beginning at the outer edge of the soma. The area under the curve of the Sholl analysis was estimated using the trapezoidal method, in Excel.

### Statistics

The two-tailed Mann-Whitney test was used to compare parameters of T neurons and non-T neurons because some parameters of non-T neurons (a heterogeneous grouping) were not normally distributed and/or had different standard deviations than T neurons. Statistical tests were performed using Instat 3 (GraphPad Software).

## Results

**Figure 1:**
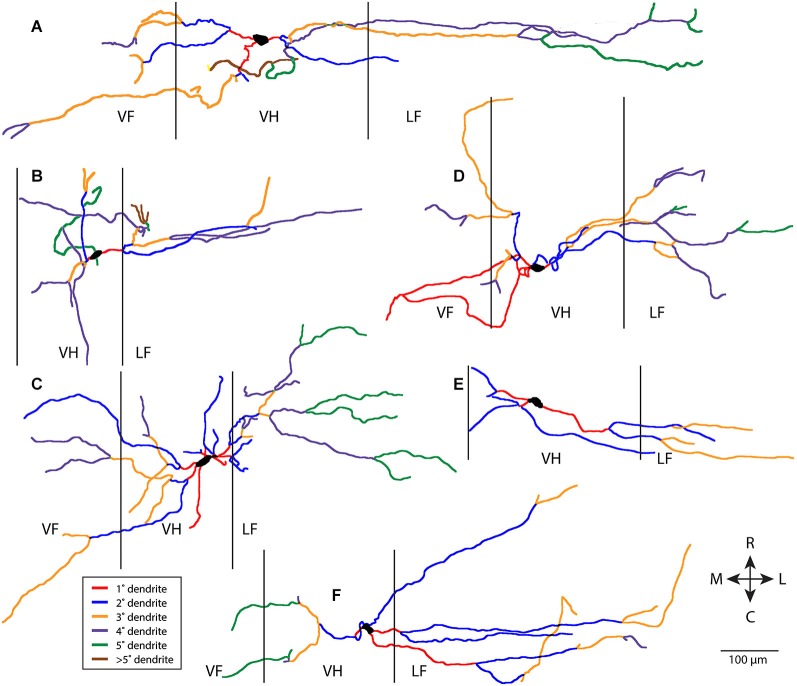
**Examples of six T neuron dendritic trees (A–F), with dendritic branches color-coded by branch order**. Vertical lines indicate borders of gray matter. 1°, primary; 2°, secondary, etc. R, rostral; C, caudal; M, medial; L, lateral; VF, ventral funiculus; VH, ventral horn; LF, lateral funiculus.

Figure 1 shows six examples of T neuron morphologies, with dendritic segments color-coded by whether they were primary, secondary, etc. These examples were chosen to illustrate both the range of dendritic characteristics of T neurons and the features T neurons have in common. T neuron dendritic trees were relatively simple. Most dendritic segments appeared to be oriented more mediolaterally than rostrocaudally. There appeared to be relatively little branching of the dendrites, even though the labeled dendrites were typically several hundred µm long. Thus, a relatively large proportion of the dendrites appeared to be primary (red, in Figure 1) and a relatively small proportion appeared to be high-order dendrites (brown for >5th-order dendrites in Figure 1).

**Figure 2:**
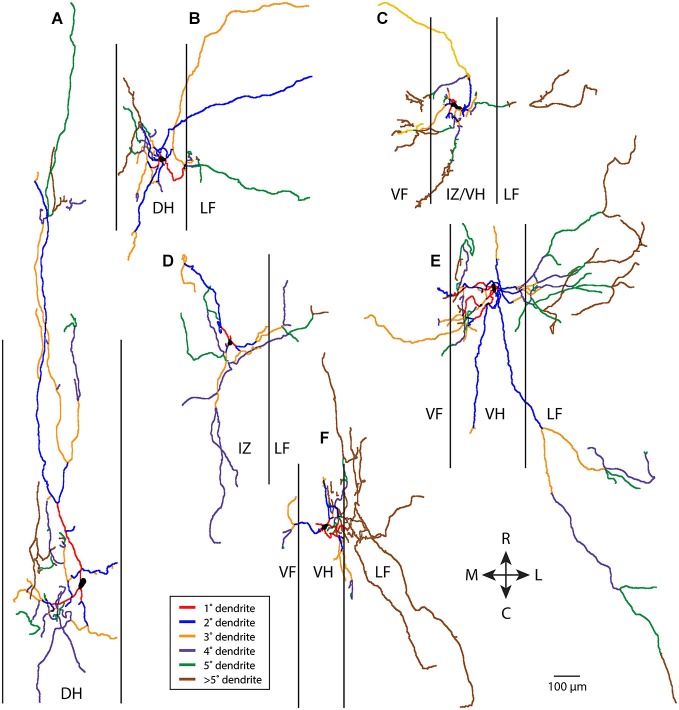
**Examples of six non-T neuron dendritic trees (A–F), with dendritic branches color-coded by branch order**. Vertical lines indicate borders of gray matter. 1°, primary; 2°, secondary, etc. R, rostral; C, caudal; M, medial; L, lateral; DH, dorsal horn; IZ, intermediate zone; VF, ventral funiculus; VH, ventral horn; LF, lateral funiculus.

In contrast, non-T neurons (a heterogeneous grouping of convenience defined by relatively greater rostrocaudal vs. mediolateral dendritic length—see Materials and Methods) typically had more complex dendritic branching. Six examples of non-T neurons are shown in Figure 2. Branching could be quite extensive and occur along multiple angles, even though many dendritic branches did not extend as far from the soma as some T neuron dendrites. A relatively small proportion of the dendrites appeared to be primary and a relatively large proportion appeared to be high-order.

**Figure 3:**
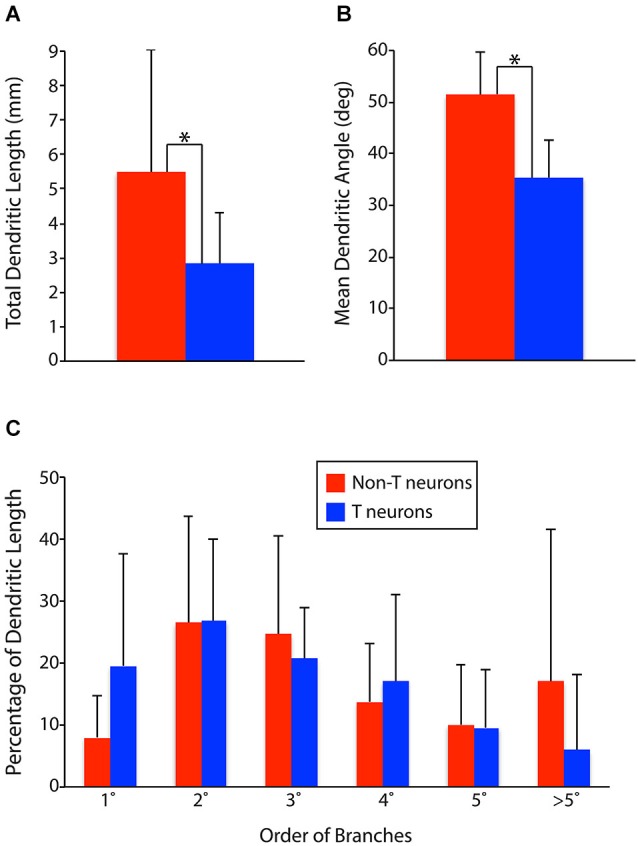
**Comparisons of dendritic parameters between T neurons (*n* = 17) and non-T neurons (*n* = 14). (A)** Total dendritic length. **(B)** Mean dendritic angle (0° = mediolateral; 90° = rostrocaudal; in horizontal sections). **(C)** Percentage of dendritic length by dendrite branch order. 1°, primary; 2°, secondary, etc. Vertical lines, SD; *, statistically significant difference.

To test these hypotheses quantitatively, we measured the total dendritic length, proportion of dendritic length taken by primary dendrites, secondary dendrites, etc., and mean dendritic angle (the mean of the angles of all the individual dendritic segments of a neuron, weighted by the proportional length of each dendritic segment) for all the T neurons and non-T neurons (Figure 3). T neurons had significantly less total dendritic length than non-T neurons (Figure 3A; *p* = 0.007). T neurons also had significantly lower [i.e., closer to mediolateral (0°), as opposed to rostrocaudal (90°)] mean dendritic angles than non-T neurons (Figure 3B; *p* < 0.0001). In addition, T neurons appeared to have a greater proportion of dendritic length in primary dendrites and a lesser proportion in >5th-order dendrites (Figure 3C), so we compared these values statistically. The T neuron percentage of dendritic length in primary dendrites was significantly higher (*p* = 0.04), but the percentage in > 5th-order dendrites was not significantly lower (*p* = 0.13). Note that our analyses collapsed the third dimension, dorsoventral, so it is possible that the neurons’ dendritic length along the dorsoventral axis differed between groups and that this difference would have altered the differences we observed in total dendritic length.

**Figure 4:**
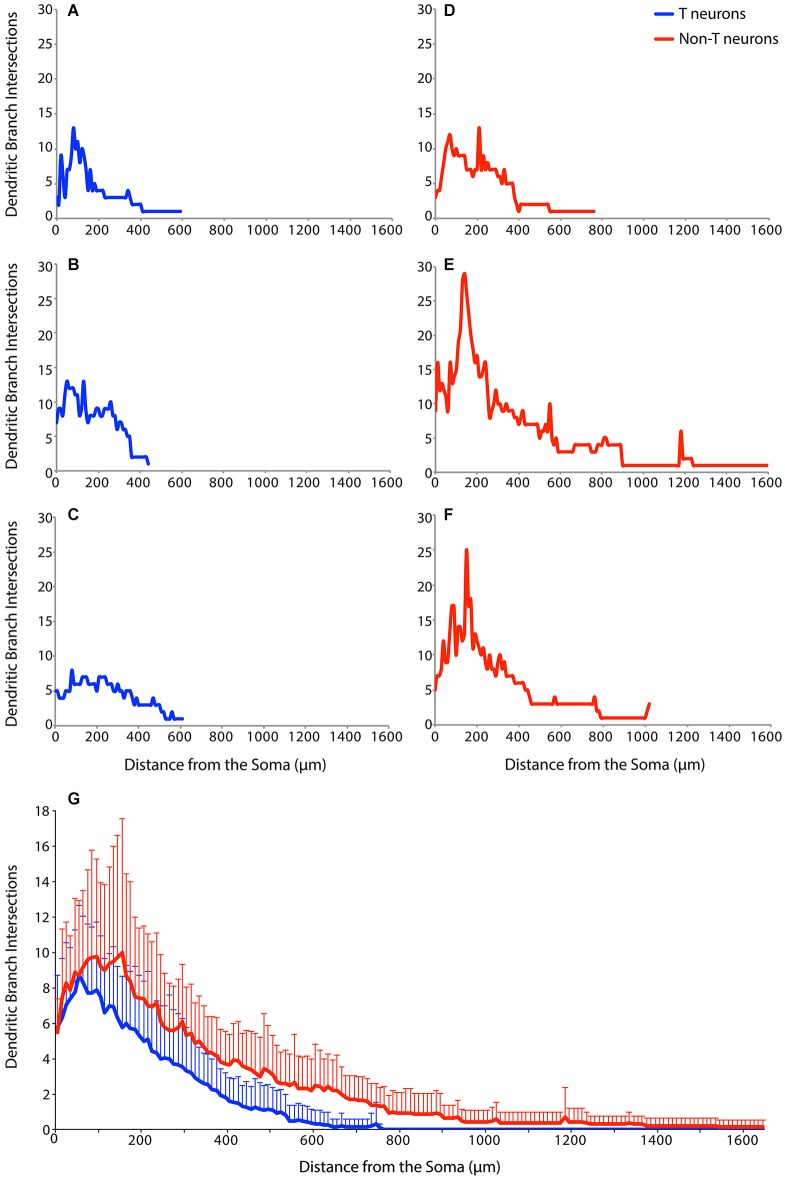
**Examples of Sholl analyses for three T neurons (A–C, blue), three non-T neurons (D–F, red), and (G) the superimposed means (+ SD) for all T neurons (blue**; ***n* = 17) and non-T neurons (red**; ***n* = 14)**. Dendritic intersections were measured every 10 µm from the soma.

An additional method of characterizing dendritic branching is via Sholl analysis, which measures the number of intersections between dendritic branches and concentric circles of increasing radii surrounding the soma (Sholl, 1953; Binley et al., 2014). Figure 4 shows Sholl analyses of example T neurons (Figures 4A–C, blue) and non-T neurons (Figures 4D–F, red). The example T neurons had a peak of dendritic branching 50–100 µm from the soma, while the non-T neurons had a broader peak and/or a second peak ~200 µm from the soma. In these examples, T neuron branching ended within 600 µm of the soma, while non-T neuron branching could continue beyond 1 mm (Figures 4E,F).

To assess dendritic branching as a function of distance from the soma over the entire sample of 17 T neurons and 14 non-T neurons, we calculated the means of the Sholl analyses for T neurons (blue) and non-T neurons (red) (Figure 4G). T neuron and non-T neuron mean dendritic branching was similar within 50 µm of the soma. But T neuron mean branching peaked at ~50 µm from the soma, while non-T neuron mean branching peaked at 100–200 µm from the soma. Following these peaks of dendritic branch intersections, the mean number of non-T neuron intersections remained ~1 intersection more than the mean number of T neuron intersections from ~200 µm through ~600 µm from the soma. T neuron mean dendritic intersections were nearly 0 beyond ~600 µm from the soma, while non-T neuron intersections continued for about twice this distance from the soma. We compared overall branching of T vs. non-T neurons using the area under the curve of the Sholl analysis, which was significantly greater (*p* = 0.0019) for non-T neurons (mean ± S*D* = 4065 ± 1958) than for T neurons (2236 ± 1100).

Mounting the horizontal tissue sections may have compressed the tissue along the dorsoventral axis for all labeled cells. If so, one would expect this effect to be larger for T neurons than for non-T neurons, because T neurons tend to have dendrites that are longer along the dorsoventral axis (and mediolateral axis) than non-T neurons, by definition. This would make dendritic branch points appear to occur closer to the soma than they actually were. Thus, this effect might reduce the proportion of total dendritic length that is in primary dendrites, especially for T neurons. Because we found that T neurons had a higher proportion of total dendritic length in primary dendrites, the actual difference between T neurons and non-T neurons in this property could be larger than what we have shown. Also, this effect would shift the total number of branch points leftward in the Sholl analyses. T neurons were found to have a similar number of branch points very near the soma (but fewer at all greater distances), so it is possible that T neurons actually have less branching than non-T neurons near the soma.

We also measured the distance dendrites extended into the white matter as a percentage of the mediolateral width of each funiculus, but there were no significant differences between T and non-T neurons in these measurements (T vs. non-T means ± SD: dorsal funiculus, 10.4 ± 29.7 vs. 11.1 ± 28.5; ventral funiculus, 31.9 ± 39.0 vs. 21.9 ± 39.4; lateral funiculus, 74.9 ± 26.8 vs. 78.4 ± 33.3; *p* > 0.3 in each case).

## Discussion

We have shown here that a class of multifunctional spinal interneurons called transverse interneurons or T neurons [defined by their lower ratios of rostrocaudal/(mediolateral + dorsoventral) dendritic extent and rostrocaudal/mediolateral soma extent] have additional quantitative morphological properties that distinguish them from other interneurons. Specifically, T neuron dendritic trees differ from those of other scratch-activated spinal interneurons in having significantly less total dendritic length, a significantly higher proportion of primary dendritic length, a mean dendritic orientation that is significantly closer to mediolateral than rostrocaudal, and less dendritic branching far from the soma. These morphological properties add to the set of physiological properties previously documented for T neurons, which include higher peak firing rates and larger membrane potential oscillations during scratching motor patterns, briefer action potentials, and briefer afterhyperpolarizations (Berkowitz et al., 2006).

Collectively, these morphological and physiological properties suggest some hypotheses about the role(s) T neurons may play in spinal motor control. Their high firing rates and large membrane potential oscillations make T neurons good candidates to be central pattern generator neurons and/or last-order premotor interneurons. Because motoneurons typically have a low input resistance and a high action potential threshold, they must be driven during locomotion and scratching either by many premotor interneurons simultaneously or by premotor interneurons that reach high firing rates rhythmically. T neurons have high peak firing rates and are rhythmically activated during both swimming and scratching (Berkowitz et al., 2006; Berkowitz, 2008), consistent with the latter possibility. At least some T neurons have ventral horn axon terminals (Berkowitz et al., 2006), which is also consistent with being last-order premotor interneurons.

The long mediolateral dendrites of T neurons, which can reach the lateral edge of the spinal cord and often reach into the ventral funiculus as well (Berkowitz et al., 2006), would allow them to receive synaptic inputs from a variety of ascending and descending axons in the lateral and ventral funiculi. Descending propriospinal axons have been shown to have swellings within the lateral funiculus (Berkowitz and Stein, 1994), suggesting that they make en passant synapses onto local dendrites. T neuron dendrites in the lateral and ventral funiculi may receive funicular axonal en passant synaptic inputs from multiple sources, including propriospinal axons from other segments and supraspinal descending axons. One might expect either central pattern generator neurons or last-order premotor neurons to integrate inputs from multiple brain and spinal cord sources that modulate motor output. The relative lack of branching of T neuron dendrites should make T neurons electrically more compact and increase the effectiveness of synaptic inputs they receive far from the soma, as would be the case for en passant synapses onto dendrites in the lateral and ventral funiculi. Thus, the long but relatively simple mediolateral dendrites of T neurons may help them serve as a kind of hub to effectively integrate inputs that modulate locomotion and scratching.

Is there a mammalian equivalent of T neurons? Adult cat lamina VII neurons receiving group II afferent input tend to have long dendrites that enter the white matter, have limited branching, and extend further mediolaterally and dorsoventrally than rostrocaudally, like T neurons; many also have ventral horn axon terminals (Bras et al., 1989). In adult lampreys, several classes of interneurons have dendrites that are more extensive mediolaterally than rostrocaudally, like T neurons, but especially “excitatory interneurons” (with ipsilateral axons that can project rostrally and/or caudally) and CC interneurons (with commissural axons that project caudally) (Buchanan, 2001). T neuron axons can be ipsilateral or contralateral and project rostrally and/or caudally (Berkowitz et al., 2006).

For species in which the spinal cord is typically studied early in development, such as zebrafish, tadpoles, chicks, and mice, the question of whether there is a class of spinal interneuron with a dendritic morphology similar to T neurons is complicated by the fact that, although axonal pathways are well defined at these ages, dendritic branching of spinal interneurons is quite limited early in development (Goulding, 2009; Grillner and Jessell, 2009). Thus, the mature dendritic morphology of such interneurons is not known. Nonetheless, the somato-dendritic morphologies of at least some mouse Shox2 interneurons, which are excitatory, have ipsilateral axons, and appear to play a role in rhythm generation, are consistent with those of T neurons (Dougherty et al., 2013).

It may be that limbed vertebrates also have additional types of spinal interneurons that do not occur in animals that only generate axial movements, such as zebrafish and hatchling tadpoles. Ideally, we would compare T neurons’ morphologies to those of mouse spinal interneurons that can be genetically identified by combinatorial expression of particular genes, especially transcription factors (Goulding, 2009; Grillner and Jessell, 2009). This may have to wait until such interneuron types can be reliably identified by expression of genes late in development or in adulthood, when dendritic trees are mature. Stable expression of some genes, including transcription factors, has been seen for some types of adult mouse brainstem neurons (Gray, 2008). This approach may in the future allow genetic identification of adult spinal cord neuronal types as well.

## Conflict of interest statement

The authors declare that the research was conducted in the absence of any commercial or financial relationships that could be construed as a potential conflict of interest.
